# Central Lines and Their Complications in Neonates: A Case Report and Literature Review

**DOI:** 10.3390/children11010026

**Published:** 2023-12-25

**Authors:** Tina Perme

**Affiliations:** Neonatal Intensive Care Unit, Department for Perinatology, Division of Gynaecology and Obstetrics, University Medical Centre Ljubljana, 1000 Ljubljana, Slovenia; tina.perme@kclj.si

**Keywords:** central lines, complications, neonate, CLABSI, pericardial effusion, cardiac tamponade, portal vein thrombosis

## Abstract

Central lines are essential devices in NICUs, used primarily in preterm neonates and critically ill term neonates. They are typically divided into non-tunnelled, tunnelled and totally implanted. In light of the increasing use of central lines in the NICU setting, monitoring of the risk factors associated with complications has to be an important part of neonatal care quality management. Presented here is a case of a preterm neonate with cardiac tamponade caused by UVC tip migration. Among complications of central lines are CLABSI, with an incidence of 3 to 21 per 1000 catheter days, and portal vein thrombosis, which is common but probably under-recognised, whereas other mechanical complications such as pericardial and pleural effusions are rare, with an incidence of less than 1%. Complications can cause injury to the neonates, as well as increase the costs of health services because of increases in the length of stay in the NICU. It is recommended that the catheter tip location is confirmed either by X-ray or ultrasonography. In order to minimise the risk of CLABSI, the use of bundles is recommended. Certain recommendations need to be followed when using different types of catheters. Future research is aimed at novel ways of central line securement to minimise mechanical complications and the use of antimicrobial catheters to reduce the rate of CLABSI.

## 1. Introduction

In Neonatal Intensive Care Units (NICUs), central lines are essential devices, particularly in preterm neonates and in term neonates who are critically ill or require surgical interventions [1]. In very low birth weight babies (VLBW), in which enteral nutrition is a challenge due to gastrointestinal immaturity, parenteral nutrition is an important modality to improve nutrition and minimise growth failure, which is associated with various short-term morbidities and poor long-term outcomes [2]. In this population of neonates, central lines are used for administering intravenous fluids and parenteral nutrition, as well as hypertonic or locally toxic medications [3]. In other neonatal populations, central lines are useful for monitoring the haemodynamic status, administering parenteral nutrition, blood products and chemotherapeutic agents, or the infusion of other fluids [4]. Although lifesaving, the use of central lines is associated with mechanical and infectious complications [5]. In this article, we will first consider different forms of central lines used in the neonatal population, describe a case of a preterm infant with a central line-associated complication, and then review the most common complications associated with central lines and try to establish some recommendations for the use and maintenance of central lines.

## 2. Central Line Types

Central lines are typically divided into non-tunnelled (centrally or peripherally inserted), tunnelled and totally implanted [5]. The latter two are of limited use in the neonatal setting [6]. Depending on the policy of each particular unit, the first central line of choice immediately after birth is an umbilical venous catheter (UVC), sometimes alongside an umbilical arterial catheter (UAC). There is no good research to date that would definitively answer the question of how long the UVC can be left in place. However, based on some best practice reports, if there is a continuous need for a UVC beyond 5–7 days, an effort should be made to replace it with another central line [7]. A less invasive alternative to a central catheter, which is usually inserted through a large vein, such as the femoral, jugular or subclavian vein, is a peripherally inserted central catheter (PICC). PICCs are fine and pliable catheters that are inserted through a peripheral vein, with the tip preferably lying in a central vein, usually the superior vena cava [8]. They were first described in the 1970s and have been used extensively ever since due to their favourable features [9].

Compared with PICCs, UVCs are less expensive and easier to insert but are of limited use because of the short duration of dwell time [3]. Despite the CDC recommendations of keeping the UVC in situ for no longer than two weeks, and in the absence of other quality evidence, most centres choose to replace the UVC after 7 days [10,11,12]. On the other hand, if longer-term access is needed, the use of PICCs seems to reduce the rate of complications, particularly if combined with a PICC maintenance team [13].

In light of the use of PICCs becoming essential in the NICU setting, monitoring of the risk factors associated with complications has to be an important part of neonatal care quality management [14]. Complications, which range from tissue (i.e., phlebitis, necrosis) to systemic (i.e., pneumothorax, cardiac tamponade, sepsis), can cause injury to the neonates, as well as increase the costs of health services because of increases in the length of stay in the NICU [15,16,17,18]. Therefore, it is important to be aware of the potential complications, clinical presentations and preventive strategies. This topic will be discussed in the following sections.

## 3. Case Presentation

The patient was a preterm newborn boy, born at a gestational age of 33 weeks +1 after a Caesarean section due to maternal placental abruption. His birth weight was 2200 g. Shortly after birth, he developed mild respiratory distress, which was treated with non-invasive mechanical ventilation. Because of suspected infection as well as trouble feeding, a UVC was inserted. The placement of UVC was corrected following an X-ray so that the tip of the UVC was outside of the right atrium. Antibiotics, as well as parenteral nutrition, were administered via the catheter. On day 4, the baby became increasingly unwell, with mottled skin colour, recurring apnoeic episodes and tachycardia. Both septic shock as well as pneumothorax were initially suspected but were ruled out. As the baby appeared in shock, a bedside echocardiography was performed, which showed pericardial effusion with signs of cardiac tamponade and cardiogenic shock; 16.5 mL of white fluid was evacuated through subxyphoid pericardial punction, and the baby’s condition rapidly improved. There was minimal effusion present on the following day, and the baby’s clinical course thereafter remained unremarkable.

In the past few years, three preterm newborns in our unit were treated for pericardial effusion caused by a central venous catheter, either UVC, PICC or non-tunnelled venous catheter, despite all catheter tip positions having been verified by X-ray. As our cases show, despite careful confirmation of the position of the catheter, pericardial effusion can still develop, and it should thus be considered a differential diagnosis in a rapidly deteriorating baby with a central venous catheter.

In the following sections, we will discuss central line complications, ways to reduce the rate of complications and future research into neonatal central lines.

## 4. Complications of Central Lines

While having central line access is vitally important in NICU patients, its presence is associated with significant morbidity and mortality [19,20]. Complications can be divided into mechanical and non-mechanical. Mechanical complications are related to the insertion technique or can be the result of narrower lumens, predisposing them to obstruction and thrombosis [1]. The main non-mechanical complication is, of course, central line-associated bloodstream infection (CLABSI) [21]. Studies on the use of PICCs in the NICU have shown that catheter-related complications occur on average in up to 30% of PICCs placed [22,23]. One study showed that the most common complication related to the use of PICC was phlebitis, followed by infection, leakage and occlusion [22]. Effusion occurred in around 2% of cases [22].

We will now review the most common types of mechanical complications, but we will also briefly discuss CLABSI.

### 4.1. Mechanical Complications of Central Lines

There are several ways in which central lines can fail mechanically, some related to the insertion technique, some to the medication used and some to the mechanical properties of the catheter itself [24]. Mechanical complications range from infiltration, occlusion, thrombosis, misplacement, and migration to breakage with subsequent extravasation and phlebitis [24,25]. Fortunately, acute life-threatening events such as pericardial effusion, cardiac tamponade and massive pleural effusion are rare and occur in infants with such catheters with an incidence of about 1–3% [26,27]. 

#### 4.1.1. Pleural Effusion

Many case reports have reported total parenteral nutrition (TPN) extravasation in the pleural space as the most common aetiology of pleural effusions as a central line complication in neonates [25,28]. The effusion is usually unilateral, with bilateral pleural effusions being very rarely described [25]. In cases of pleural effusions in neonates with central lines, TPN extravasation should be carefully differentiated from a chylothorax due to a completely different course of management [25]. There are several mechanisms of central line-related pleural effusion that have been proposed over the years [25]. Among them are vessel perforation or retrograde flow passage through the lymphatic duct induced by catheterisation, mechanical vein erosion and hyperosmotic injury of the endothelium, which increases permeability and the risk of thrombosis of the pulmonary vessels [25,29]. While pleural effusion associated with the presence of a central line is a relatively rare complication, it has been described with a frequency of 0.4 per 1000 catheter days for a PICC line, while only four cases have been described associated with a UVC [28]. Misplacement of PICC during insertion has usually been reported as the aetiology [30]. Premature infants might be more inclined to vessel wall erosion, especially when PICCs are positioned in the upper extremities [29]. Frequent arm movements can also be problematic in that they can change the position of the catheter tip, thus increasing the risk of superior vena cava erosion when administering hyperosmolar fluid [25]. 

#### 4.1.2. Pericardial Effusion

One of the most severe and life-threatening complications of a central line is an effusion of fluids in the pericardial sac with consequent cardiac tamponade [8,31]. The incidence of pericardial effusion and cardiac tamponade is about 0.07–2%; however, mortality can be as high as 75% without pericardiocentesis and only around 8% when pericardiocentesis is performed [32,33]. One of the reasons why pericardial effusion is more common in preterm and term neonates is that the thin wall of the heart is more easily damaged, with myocardium even being absent in some sections of the atrial wall [34]. In addition to the weakness of the myocardium wall, there is also greater fragility in the vessel walls, as evidenced by the fact that pericardial effusions generally occur in smaller infants and in infants of lower gestational ages, whereas pericardial effusion in older children and especially adults is much less likely [8,35,36]. Perforation of the myocardium can occur at the time of cannulation, either directly or secondary to wire use [34]. However, this seems to be an exception, and the more common cause of pericardial effusion is actually secondary to endocardial damage from repeated contact of the catheter tip with the myocardium, which results in the formation of a thrombus and adherence of the catheter tip to the myocardial wall. This brings the hyperosmolar fluid in direct contact with the myocardial wall, resulting in osmotic injury [34]. Interestingly, the lateral wall of the superior vena cava seems to be more fragile than the medial wall, meaning there is an increased risk of erosion when the tip of the catheter abuts the lateral wall or creates an acute angle [8,37]. While erosion of the vessel wall can occur directly from the force of the jet, it can also occur without prior trauma just from the nature of the infusate [8,37]. In particular, total parenteral nutrition seems to present the highest risk of damaging the vessel and cardiac wall, leading to pericardial effusion [34,38,39].

#### 4.1.3. Portal Vein Thrombosis

Previously, portal vein thrombosis has been considered a rare occurrence; however, with the increasing use of routine ultrasound, it has been recognised more commonly, with incidences of between 0 and 50% reported in the literature [40,41]. In addition to the insertion of a UVC, risk factors include the infusate composition, low birth weight, hypoxia, sepsis and congenital malformations, among others [42]. Most thrombi spontaneously resolve; however, portal vein thrombosis is an important cause of portal hypertension later in childhood [43]. The highest rate of complications is seen if the tip of the UVC is placed in the left portal vein, and there is no particular risk if the UVC is placed outside of the portal system [42]. Even if UVCs are initially placed appropriately, they can migrate into the portal vein, which is why regular ultrasound monitoring of tip position is important to reduce the risk of a potentially severe complication [41].

#### 4.1.4. Thrombosis

Neonates, in general, have the highest risk of thromboembolic complications, and this is especially true in critically ill children [44]. The most important risk factor for thrombosis in neonates is the presence of a central catheter [45]. Additionally, there are several risk factors that predispose neonates with inserted central lines to thrombosis, such as low birth weight, prematurity, prolonged dwell time of more than 6 days, UVC malplacement, mechanical ventilation, surgery and multiple vein punctures [44,46,47]. The incidence of thrombosis associated with PICC lines has been cited in the literature as between 0.3 and 28.3% [48]. On the other hand, thrombosis has been described in 75% of children with UVCs [49]. However, these thrombi are usually asymptomatic and resolve spontaneously, which is why routine ultrasound screening is not recommended [49]. When installed, the central line promotes thrombosis by increasing the absorption of proteins, adhesion of leukocytes and platelets, and increasing thrombin production [45]. Another important mechanism of thrombosis in neonates with central lines is CLABSI, which will be discussed in the following section [50].

### 4.2. Central Line-Associated Bloodstream Infection (CLABSI)

CLABSI is defined as a bloodstream infection confirmed by a laboratory, not related to an infection of another site, and that occurs within 48 h of catheter placement or removal [51]. The most common causative organism is coagulase-negative staphylococcus. CLABSI is a major cause of morbidity and mortality in the NICU setting [52]. Preterm neonates are especially susceptible due to their poor skin integrity, an increased need for invasive procedures, immature immunity and prolonged stay in the NICU [53]. The incidence of CLABSI in neonates with UVCs is 5–10%, with the highest rates occurring in neonates weighing less than 1500 g [11,54]. Despite its risks, obtaining a central line is vital for the survival of these babies, which is why there is a continuous strive towards a zero rate of CLABSI [52]. Ways of achieving that will be discussed in the following section on recommendations for the use of central lines.

Typical complications related to central lines in neonates are summarised in Table 1.

## 5. Recommendations for Reducing the Rate of Central Line Complications in Neonates

### 5.1. Reducing the Rate of Mechanical Complications

In order to decrease the risk of potentially life-threatening mechanical complications, proper positioning of the catheter is recommended [57]. The tip of the UVC should be at the junction of the inferior vena cava and right atrium, while the tip of the PICC should be just proximal to the entry into the right atrium [58,59]. More specifically, the satisfactory position of a UVC is within 0.5 cm of the inferior vena cava and right atrium junction, while the satisfactory position of a PICC is in the superior or inferior vena cava, within 0.5 cm of the junction of the right atrium and either of the vena cava [57]. The current standard for line tip positioning is still conventional thoraco–abdominal radiography, which estimates tip position based on static landmarks, such as cardiac silhouette or the diaphragm contour [57]. When confirming the tip position, it is also important to know how different factors can affect the position of the tip. Migration of UVC after insertion has been widely documented, in part due to the drying of the Wharton jelly, which causes inward migration, while outward migration has been attributed to the gradual distension of the abdomen as the bowel begins to fill with gas [58]. When confirming the tip position of the PICC, it is important to know how it can be affected by the arm positioning. The maximum movement of the catheter toward the heart, when inserted in the basilic vein, is when the arm is adducted and elbow is in the flexed position; if PICC is in the cephalic vein, the tip is closest to the heart when the arm is abducted and the elbow in the flexed position; and if PICC is in the axillary vein, the tip is closest to the heart when the arm is adducted regardless of the position of the elbow [60]. When taking radiographs to confirm tip positions, the position of the arm should be adjusted accordingly [60]. However, ultrasonography has recently been suggested as the better method because of its speed, safety and accuracy; its radiation-free nature is particularly important in the neonatal period. Ultrasonography is also very practical because tip migration may frequently occur, and following the location of the catheter tip over time is thus recommended [61,62]. Tip migration seems to be an important risk factor for a mechanical complication [8]. Possible mechanisms for outward migration include loosening of the dressing, movement of the PICC while changing the dressing, and growth of the neonate [8]. It is therefore important that central lines are not only carefully inserted but that dressings are changed carefully and frequently and that catheter tip locations are frequently confirmed, preferably using ultrasonography [24,58].

There has been significant research into using low molecular weight heparin (LMWH) as a prophylaxis against central catheter-related thrombosis. However, there is still insufficient evidence to recommend LMWH as thromboprophylaxis in neonates with central catheters [5].

### 5.2. Reducing the Rate of Infective Complications

A proven way to reduce the rate of CLABSI in neonates with central lines is through the use of bundles. A bundle is a combination of evidence-based procedures that, if adhered to correctly and stringently, have been shown to improve outcomes [63]. Important parts of such bundles are hand hygiene, the material of central lines and care of the central dressing, how drugs are prepared and administered, optimising or minimising dwell times, creating and using checklists and having a dedicated and specialised team for line insertion and maintenance [52]. One of the most important risk factors for any complication, but especially for the development of CLABSI, is, of course, dwell time. It is recommended that the central line should be removed as soon as enteral nutrition reaches 120 mL/kg/day, with some authors suggesting removal when a feeding volume of 100 mL/kg/day is reached [52,64].

## 6. Future Research into Reducing Complications

The most efficient way to avoid central line complications is to not use them at all. However, this is an unrealistic goal to strive towards in the NICU setting, but there is considerable research ongoing into new procedures that could help in reducing the rates of complications.

Securing a UVC is difficult as it is the only catheter that is inserted into an area of tissue that is going to necrose in the subsequent days. With the standard securing techniques, allowing for open ventilation and umbilical stump desiccation, the insertion site is prone to accidental contact and inadvertent bacterial inoculation [54]. There has been interesting research into using dome-shaped silicone devices that would not only secure the UVC in place but also introduce a physical barrier that would reduce the risk of bacterial inoculation [54]. Research is, of course, ongoing.

On the other hand, properly securing a PICC is also vitally important, as PICCs in neonates are prone to dislodgement, thus leading to mechanical complications. Recently, there has been a considerable amount of research into using a drop of cyanoacrylate glue at the PICC insertion point [65]. As the glue is transparent, the insertion point remains visible at all times. In one study, by using glue, they were able to reduce the rate of mechanical complications with no adverse skin reactions [65]. In addition, there is a randomised controlled trial in progress, looking at the use of cyanoacrylate glue for securing UVCs [58]. The glue could work in concert with other securement methods as it may provide an immediate haemostatic effect. 

Finally, one way of reducing the rate of CLABSI is the use of a new type of antimicrobial catheter, which is coated with an antibiotic (rifampicin) and an antifungal (miconazole) and is currently undergoing several randomised controlled trials [9].

## 7. Recommendations for Using Different Types of Catheters

Each NICU has its own guidelines on when and how to use different types of catheters. However, there are some universal recommendations that should be taken into account when deciding on which type of catheter to use.

### 7.1. Umbilical Arterial Catheter

Umbilical arterial catheters provide direct access to the arterial system. The primary indications for using UACs are the need for frequent blood sampling, measuring arterial blood gases or continuous blood pressure monitoring [66]. Some of the groups of neonates who benefit from the use of UACs are very low birth weight infants, preterm infants on mechanical ventilatory support, neonates with hypoxic–ischaemic encephalopathy undergoing therapeutic hypothermia and other critically ill neonates [66]. There is an increased risk of infection and thrombosis with prolonged dwell time, which is why the necessity of the UAC needs to be evaluated daily and continuation of its use beyond five days is not recommended [66].

### 7.2. Umbilical Venous Catheter

The indication for umbilical vein catheterisation is the need for IV access in newborn resuscitation, for blood transfusions or as a short-term venous access [67]. Additionally, according to some guidelines, UVCs should be used in newborns with gestational ages 28 weeks or less, newborns of gestational ages of more than 29 weeks who are mechanically ventilated and require FiO_2_ of more than 40% and in newborns of gestational ages of more than 29 weeks who are haemodynamically unstable [68]. UVCs with a calibre of 3.5 Fr are recommended for infants weighing less than 3.5 kg, while a calibre of 5 Fr should be used in infants weighing more than 3.5 kg [58]. If simultaneous administration of incompatible solutions is anticipated, double or triple-lumen catheters can be used [58]. In addition, the use of multi-lumen catheters is recommended in low birth weight infants, as it may reduce the need for additional peripheral vascular access [58].

### 7.3. Peripherally Inserted Central Venous Catheter

PICCs are inserted through a direct puncture of a superficial peripheral vein. They are especially useful for providing parenteral nutrition, long-term intravenous therapy and antibiotic therapy [6]. Their main disadvantages are that they are not appropriate for venous blood sampling, and they cannot be used for central venous pressure monitoring [6]. There are also double-lumen PICCs available if multiple infusions are anticipated, and additional IV access is difficult to obtain.

## 8. Conclusions

Central lines are important tools in the NICU to ensure the survival of the most vulnerable and premature neonates. However, they can themselves contribute to morbidity and mortality if not used judicially. It is important to assess if a central line is needed in the first place, minimise dwell time, use a central line maintenance team and protocol to reduce the rate of CLABSI and periodically confirm the catheter tip to avoid mechanical complications. Future advances in ways of securing catheters and minimising the risks of infection are, of course, highly anticipated.

## Figures and Tables

**Table 1:** Incidences of typical complications in relation to typical catheters.

Type of Complication	Typical Catheter Type	Risk Factors	Incidence (%)	Incidence (per 1000 Catheter Days)
CLABSI	UVC	low birth weight, dwell time		3.2–21.8 [55]
	UVA		NA	
	PICC			
Pleural effusion	UVA	NA	NA	NA
	UVC	TPN, tip migration	4 cases	NA
	PICC	TPN, more distal tip	0.29 [8]	0.4 [28]
Pericardial effusion	UVC PICC	tip migration TPN, more proximal tip	0.22–0.67 [56]	NA
Portal vein thrombosis	UVC	low birth weight, dwell time, infusate, sepsis	0–50 [41]	NA
Thrombosis	UVC	dwell time	75 [49]	NA
	PICC	sepsis	0.3–28.3 [48]	NA

NA—not available.

## References

[1] Soares B.N., Pissarra S., Rouxinol-Dias A.L., Costa S., Guimaraes H. (2018). Complications of central lines in neonates admitted to a level III Neonatal Intensive Care Unit. J. Matern. Fetal Neonatal Med..

[2] Harding J.E., Cormack B.E., Alexander T., Alsweiler J.M., Bloomfield F.H. (2017). Advances in nutrition of the newborn infant. Lancet.

[3] Gupta S., Patwardhan G., Parikh T., Kadam S., Vaidya U., Pandit A. (2021). Which long line do we use in very low birth weight neonates; umbilical venous catheter or peripherally inserted central catheter?. J. Neonatal Perinat. Med..

[4] Klerk C.P., Smorenburg S.M., Buller H.R. (2003). Thrombosis prophylaxis in patient populations with a central venous catheter: A systematic review. Arch. Intern. Med..

[5] Pelland-Marcotte M.C., Amiri N., Avila M.L., Brandao L.R. (2020). Low molecular weight heparin for prevention of central venous catheter-related thrombosis in children. Cochrane Database Syst. Rev..

[6] Bayoumi M.A.A., van Rens R., Chandra P., Shaltout D., Gad A., Elmalik E.E., Hammoudeh S. (2022). Peripherally inserted central catheters versus non-tunnelled ultrasound-guided central venous catheters in newborns: A retrospective observational study. BMJ Open.

[7] Keir A., Giesinger R., Dunn M. (2014). How long should umbilical venous catheters remain in place in neonates who require long-term (≥5–7 days) central venous access?. J. Paediatr. Child Health.

[8] Sertic A.J., Connolly B.L., Temple M.J., Parra D.A., Amaral J.G., Lee K.S. (2018). Perforations associated with peripherally inserted central catheters in a neonatal population. Pediatr. Radiol..

[9] Barone G., Pittiruti M. (2020). Epicutaneo-caval catheters in neonates: New insights and new suggestions from the recent literature. J. Vasc. Access.

[10] Ainsworth S., McGuire W. (2015). Percutaneous central venous catheters versus peripheral cannulae for delivery of parenteral nutrition in neonates. Cochrane Database Syst. Rev..

[11] O’Grady N.P., Alexander M., Burns L.A., Dellinger E.P., Garland J., Heard S.O., Lipsett P.A., Masur H., Mermel L.A., Pearson M.L. (2011). Guidelines for the prevention of intravascular catheter-related infections. Clin. Infect. Dis..

[12] Hess S., Poryo M., Ruckes C., Papan C., Ehrlich A., Ebrahimi-Fakhari D., Bay J.S., Wagenpfeil S., Simon A., Meyer S. (2023). Assessment of an umbilical venous catheter dwell-time of 8–14 days versus 1–7 days in very low birth weight infacts (UVC—You Will See): A pilot single-center, randomized controlled trial. Early Hum. Dev..

[13] Butler-O’Hara M., D’Angio C.T., Hoey H., Stevens T.P. (2012). An evidence-based catheter bundle alters central venous catheter strategy in newborn infants. J. Pediatr..

[14] Sarmento Diniz E.R., de Medeiros K.S., Rosendo da Silva R.A., Cobucci R.N., Roncalli A.G. (2021). Prevalence of complications associated with the use of a peripherally inserted central catheter in newborns: A systematic review protocol. PLoS ONE.

[15] Li R., Cao X., Shi T., Xiong L. (2019). Application of peripherally inserted central catheters in critically ill newborns experience from a neonatal intensive care unit. Medicine.

[16] Panagiotounakou P., Antonogeorgos G., Gounari E., Papadakis S., Labadaridis J., Gounaris A.K. (2014). Peripherally inserted central venous catheters: Frequency of complications in premature newborn depends on the insertion site. J. Perinatol..

[17] Singh A., Bajpai M., Panda S.S., Jana M. (2014). Complications of peripherally inserted central venous catheters in neonates: Lesson learned over 2 years in a tertiary care centre in India. Afr. J. Paediatr. Surg..

[18] Yu X., Yue S., Wang M., Cao C., Liao Z., Ding Y., Huang J., Li W. (2018). Risk Factors Related to Peripherally Inserted Central Venous Catheter Nonselective Removal in Neonates. BioMed Res. Int..

[19] Greenberg R.G., Cochran K.M., Smith P.B., Edson B.S., Schulman J., Lee H.C., Govindaswami B., Pantoja A., Hardy D., Curran J. (2015). Effect of Catheter Dwell Time on Risk of Central Line-Associated Bloodstream Infection in Infants. Pediatrics.

[20] Hermansen M.C., Hermansen M.G. (2005). Intravascular catheter complications in the neonatal intensive care unit. Clin. Perinatol..

[21] Callejas A., Osiovich H., Ting J.Y. (2016). Use of peripherally inserted central catheters (PICC) via scalp veins in neonates. J. Matern. Fetal Neonatal Med..

[22] Colacchio K., Deng Y., Northrup V., Bizzarro M.J. (2012). Complications associated with central and non-central venous catheters in a neonatal intensive care unit. J. Perinatol..

[23] Liu H., Han T., Zheng Y., Tong X., Piao M., Zhang H. (2009). Analysis of complication rates and reasons for nonelective removal of PICCs in neonatal intensive care unit preterm infants. J. Infus. Nurs..

[24] Hugill K., van Rens M. (2020). Inserting central lines via the peripheral circulation in neonates. Br. J. Nurs..

[25] Alhatem A., Estrella Y., Jones A., Algarrahi K., Fofah O., Heller D.S. (2021). Percutaneous Route of Life: Chylothorax or Total Parenteral Nutrition-Related Bilateral Pleural Effusion in a Neonate?. Fetal Pediatr. Pathol..

[26] Sehgal A., Cook V., Dunn M. (2007). Pericardial effusion associated with an appropriately placed umbilical venous catheter. J. Perinatol..

[27] Wrightson D.D. (2013). Peripherally inserted central catheter complications in neonates with upper versus lower extremity insertion sites. Adv. Neonatal Care.

[28] Bashir R.A., Callejas A.M., Osiovich H.C., Ting J.Y. (2017). Percutaneously Inserted Central Catheter-Related Pleural Effusion in a Level III Neonatal Intensive Care Unit: A 5-Year Review (2008–2012). JPEN J. Parenter. Enter. Nutr..

[29] Blackwood B.P., Farrow K.N., Kim S., Hunter C.J. (2016). Peripherally Inserted Central Catheters Complicated by Vascular Erosion in Neonates. JPEN J. Parenter. Enter. Nutr..

[30] Kumar S., de Lusignan S., McGovern A., Correa A., Hriskova M., Gatenby P., Jones S., Goldsmith D., Camm A.J. (2018). Ischaemic stroke, haemorrhage, and mortality in older patients with chronic kidney disease newly started on anticoagulation for atrial fibrillation: A population based study from UK primary care. BMJ.

[31] Beardsall K., White D.K., Pinto E.M., Kelsall A.W. (2003). Pericardial effusion and cardiac tamponade as complications of neonatal long lines: Are they really a problem?. Arch. Dis. Child. Fetal Neonatal Ed..

[32] Atmawidjaja R.W., Azri M., Ismail I.H. (2016). Cardiac tamponade: A rare but preventable complication of central venous catheter in neonates. Med. J. Malays..

[33] Pizzuti A., Parodi E., Abbondi P., Frigerio M. (2010). Cardiac tamponade and successful pericardiocentesis in an extremely low birth weight neonate with percutaneously inserted central venous line: A case report. Cases J..

[34] Nowlen T.T., Rosenthal G.L., Johnson G.L., Tom D.J., Vargo T.A. (2002). Pericardial effusion and tamponade in infants with central catheters. Pediatrics.

[35] Booth S.A., Norton B., Mulvey D.A. (2001). Central venous catheterization and fatal cardiac tamponade. Br. J. Anaesth..

[36] Orme R.M., McSwiney M.M., Chamberlain-Webber R.F. (2007). Fatal cardiac tamponade as a result of a peripherally inserted central venous catheter: A case report and review of the literature. Br. J. Anaesth..

[37] Menon G. (2003). Neonatal long lines. Arch. Dis. Child. Fetal Neonatal Ed..

[38] Clark E., Giambra B.K., Hingl J., Doellman D., Tofani B., Johnson N. (2013). Reducing risk of harm from extravasation: A 3-tiered evidence-based list of pediatric peripheral intravenous infusates. J. Infus. Nurs..

[39] Pezzati M., Filippi L., Chiti G., Dani C., Rossi S., Bertini G., Rubaltelli F.F. (2004). Central venous catheters and cardiac tamponade in preterm infants. Intensive Care Med..

[40] Williams S., Chan A.K. (2011). Neonatal portal vein thrombosis: Diagnosis and management. Semin. Fetal Neonatal Med..

[41] Bersani I., Piersigilli F., Iacona G., Savarese I., Campi F., Dotta A., Auriti C., Di Stasio E., Garcovich M. (2021). Incidence of umbilical vein catheter-associated thrombosis of the portal system: A systematic review and meta-analysis. World J. Hepatol..

[42] Selvam S., Humphrey T., Woodley H., English S., Kraft J.K. (2018). Sonographic features of umbilical catheter-related complications. Pediatr. Radiol..

[43] Morag I., Shah P.S., Epelman M., Daneman A., Strauss T., Moore A.M. (2011). Childhood outcomes of neonates diagnosed with portal vein thrombosis. J. Paediatr. Child Health.

[44] Haley K.M. (2017). Neonatal Venous Thromboembolism. Front. Pediatr..

[45] Makatsariya A., Bitsadze V., Khizroeva J., Vorobev A., Makatsariya N., Egorova E., Mischenko A., Mashkova T., Antonova A. (2022). Neonatal thrombosis. J. Matern. Fetal Neonatal Med..

[46] Sirachainan N., Limrungsikul A., Chuansumrit A., Nuntnarumit P., Thampratankul L., Wangruangsathit S., Sasanakul W., Kadegasem P. (2018). Incidences, risk factors and outcomes of neonatal thromboembolism. J. Matern. Fetal Neonatal Med..

[47] Koksoy C., Kuzu A., Erden I., Akkaya A. (1995). The risk factors in central venous catheter-related thrombosis. Aust. N. Z. J. Surg..

[48] Zhu W., Zhang H., Xing Y. (2022). Clinical Characteristics of Venous Thrombosis Associated with Peripherally Inserted Central Venous Catheter in Premature Infants. Children.

[49] Dubbink-Verheij G.H., Visser R., Roest A.A., van Ommen C.H., Te Pas A.B., Lopriore E. (2020). Thrombosis after umbilical venous catheterisation: Prospective study with serial ultrasound. Arch. Dis. Child. Fetal Neonatal Ed..

[50] Marquez A., Shabanova V., Faustino E.V., Northeast Pediatric Critical Care Research C. (2016). Prediction of Catheter-Associated Thrombosis in Critically Ill Children. Pediatr. Crit. Care Med..

[51] Horan T.C., Andrus M., Dudeck M.A. (2008). CDC/NHSN surveillance definition of health care-associated infection and criteria for specific types of infections in the acute care setting. Am. J. Infect. Control.

[52] Bierlaire S., Danhaive O., Carkeek K., Piersigilli F. (2021). How to minimize central line-associated bloodstream infections in a neonatal intensive care unit: A quality improvement intervention based on a retrospective analysis and the adoption of an evidence-based bundle. Eur. J. Pediatr..

[53] Payne V., Hall M., Prieto J., Johnson M. (2018). Care bundles to reduce central line-associated bloodstream infections in the neonatal unit: A systematic review and meta-analysis. Arch. Dis. Child. Fetal Neonatal Ed..

[54] Wood L.S.Y., Fuerch J.H., Dambkowski C.L., Chehab E.F., Torres S., Shih J.D., Venook R., Wall J.K. (2021). Novel Neonatal Umbilical Catheter Protection and Stabilization Device in In vitro Model of Catheterized Human Umbilical Cords: Effect of Material and Venting on Bacterial Colonization. Am. J. Perinatol..

[55] Hightower H.B., Young J.A., Thomas J., Smith J.J., Hobby-Noland D., Palombo G., McCaskey M., Benton B., Hutto C., Coghill C. (2022). Reduction of Central-line–Associated Bloodstream Infections in a Tertiary Neonatal Intensive Care Unit through Simulation Education. Pediatr. Qual. Saf..

[56] Wang J., Wang Q., Liu Y., Lin Z., Janjua M.U., Peng J., Du J. (2022). The incidence and mortality rate of catheter-related neonatal pericardial effusion: A meta-analysis. Medicine.

[57] Rossi S., Jogeesvaran K.H., Matu E., Khan H., Grande E., Meau-Petit V. (2022). Point-of-care ultrasound for neonatal central catheter positioning: Impact on X-rays and line tip position accuracy. Eur. J. Pediatr..

[58] D’Andrea V., Prontera G., Rubortone S.A., Pezza L., Pinna G., Barone G., Pittiruti M., Vento G. (2021). Umbilical Venous Catheter Update: A Narrative Review Including Ultrasound and Training. Front. Pediatr..

[59] Nourzaie R., Abbas H., Parthipun A., Boolkah S., Ahmed I., Gkoutzios P., Moser S., Monzon L., Karunanithy N., Diamantopoulos A. (2023). Atypical use of PICC as centrally inserted central catheter in infants and neonates: Report of a 10-year experience. J. Vasc. Access.

[60] Nadroo A.M., Glass R.B., Lin J., Green R.S., Holzman I.R. (2002). Changes in upper extremity position cause migration of peripherally inserted central catheters in neonates. Pediatrics.

[61] Katheria A.C., Fleming S.E., Kim J.H. (2013). A randomized controlled trial of ultrasound-guided peripherally inserted central catheters compared with standard radiograph in neonates. J. Perinatol..

[62] Michel F., Brevaut-Malaty V., Pasquali R., Thomachot L., Vialet R., Hassid S., Nicaise C., Martin C., Panuel M. (2012). Comparison of ultrasound and X-ray in determining the position of umbilical venous catheters. Resuscitation.

[63] Bannatyne M., Smith J., Panda M., Abdel-Latif M.E., Chaudhari T. (2018). Retrospective Cohort Analysis of Central Line Associated Blood Stream Infection following Introduction of a Central Line Bundle in a Neonatal Intensive Care Unit. Int. J. Pediatr..

[64] Erdei C., McAvoy L.L., Gupta M., Pereira S., McGowan E.C. (2015). Is zero central line-associated bloodstream infection rate sustainable? A 5-year perspective. Pediatrics.

[65] Piersigilli F., Iacona G., Yazami S., Carkeek K., Hocq C., Auriti C., Danhaive O. (2023). Cyanoacrylate glue as part of a new bundle to decrease neonatal PICC-related complications. Eur. J. Pediatr..

[66] Dumpa V., Avulakunta I.D. (2023). Umbilical Artery Catheterization. StatPearls.

[67] Lewis K., Spirnak P.W. (2023). Umbilical Vein Catheterization. StatPearls.

[68] Shahid S., Dutta S., Symington A., Shivananda S., McMaster University N. (2014). Standardizing umbilical catheter usage in preterm infants. Pediatrics.

